# Trends in healthcare utilization by patients with gout: A cross-sectional study using Health Insurance Review and Assessment Service data

**DOI:** 10.1097/MD.0000000000036436

**Published:** 2024-02-16

**Authors:** Do-Hyun Kang, Yoon Jae Lee, In-Hyuk Ha, Ho Seub Song, Ye-Seul Lee

**Affiliations:** a Jaseng Hospital of Korean Medicine, Seoul, Republic of Korea; b Jaseng Spine and Joint Research Institute, Jaseng Medical Foundation, Seoul, Republic of Korea; c Department of Acupuncture and Moxibustion, College of Korean Medicine, Gachon University, Seongnam, Republic of Korea.

**Keywords:** cost of care, gout, HIRA Claims Data, hyperuricemia, medical service utilization, seasonality, South Korea

## Abstract

This study aimed to analyze the distribution of gout patients and the utilization of healthcare services in South Korea to provide valuable recommendations to clinicians and policymakers. A cross-sectional study was conducted. Claims data from the Health Insurance Review and Assessment Service spanning 2010 to 2019 were utilized, and a sample of 69,680 patients was included in the study. The incidence of gout was observed to be high in male patients over the age of 40, with most patients receiving outpatient care for gout management. Nonsteroidal anti-inflammatory drugs and urate-lowering agents were the most frequently prescribed medications, with prescriptions for colchicine and febuxostat increasing among urate-lowering agents. Musculoskeletal disorders were found to be the most common comorbidities among gout patients. Although the total costs of gout management increased, there was no significant increase in cost per patient. This study provides insights into the current state of healthcare utilization for gout patients in South Korea and trends in the disease burden and use of medications. The findings have crucial implications for clinicians and policymakers involved in decision-making regarding the management and treatment of gout.

## 1. Introduction

Gout is an acute condition characterized by sterile inflammation triggered by interactions between monosodium urate crystals and the surrounding tissue environment. The pathogenesis of hyperuricemia, a precursor to gout, has been associated with several factors, including consuming purine-rich foods, alcohol intake, and renal impairment. Approximately 5% of patients with hyperuricemia exhibit serum uric acid levels above 9 mg/dL and eventually develop gout.^[1]^ Gout is a condition that can alternate between periods without symptoms and episodes of acute joint inflammation. Typically, gout affects only one joint at a time. Research on gout inflammation mechanisms has demonstrated that tissue-resident macrophages or monocytes respond to crystal deposition by phagocytosing monosodium urate crystals.^[2]^ If left untreated, urate deposits can accumulate in soft tissues, leading to recurrent arthritis attacks that affect multiple joints and cause progressive joint destruction.^[3]^

Recent reports on the prevalence and incidence of gout have produced varying results, depending on the study population and research methodology. However, studies consistently report a prevalence ranging from <1% to 6.8% and an incidence rate of 0.58 to 2.89 per 1000 person-years.^[4]^ Research on gout epidemiology has demonstrated that over the past 25 years, there has been a significant increase in the incidence, prevalence, and associated health burden of gout.^[5]^ A study using data from the national health claims database in South Korea found that the prevalence of gout increased from 3.49 (95% confidence interval [CI] 3.48–3.51) per 1000 persons in 2007 to 7.58 (95% CI, 7.55–7.60) per 1000 persons in 2015. The incidence of gout was 1.52 (95% CI, 1.51–1.53) per 1000 persons in 2009 and increased to 1.94 (95% CI, 1.93–1.95) per 1000 persons in 2015.^[6]^

The treatment of gout varies depending on the severity of symptoms. Acute gout flares are characterized by a rapid onset of severe pain within hours and are typically managed using anti-inflammatory agents such as nonsteroidal anti-inflammatory drugs (NSAIDs), glucocorticoids, and colchicine. Urate-lowering treatments (ULTs) are primarily used for long-term management in cases of chronic gout or recurrent acute flares. However, healthcare professionals generally agree that ULTs are not recommended for patients with hypertension, kidney disease, metabolic syndrome, or diabetes.^[6]^ Considering the high comorbidities of metabolic disorders among gout patients,^[7]^ lifestyle interventions such as dietary modification, restriction of alcohol consumption, and obesity management are often considered necessary,^[8]^ along with medications such as allopurinol or febuxostat.

This study aims to examine the current state of healthcare utilization for patients with gout and analyze trends over time using 10 years of claims data from the Health Insurance Review and Assessment Service (HIRA) in South Korea, covering the period from 2010 to 2019. While recent studies have examined age- and sex-specific healthcare utilization in South Korea,^[9]^ few previous studies have analyzed detailed trends in this area. By investigating seasonal trends in gout prevalence and healthcare utilization, as well as the trend in gout medications over time, this study results are expected to provide a foundation for developing policies related to health insurance schemes for chronic disease patients with gout to improve patient access to healthcare.

## 2. Methods

### 2.1. Data source

This cross-sectional study was based on data from the HIRA-National Patient Sample (HIRA-NPS), which the HIRA provided for January 2010 to December 2019. The HIRA database is constructed using claims data from medical institutions and is a useful tool for healthcare research, as it contains records of diagnoses, treatments, procedures, surgical histories, and prescribed medications. It covers 98% of the South Korean population, making it suitable for understanding the status of healthcare utilization for the entire patient population in South Korea.

The HIRA-NPS data used in this study are secondary, with all personal information and information related to corporate bodies being deidentified. The data were extracted using a stratified randomized sampling method, wherein the sampled population was stratified into age and sex groups. The data for 1 year from the date of starting medical care in the corresponding year were used in this study, with 3% of the total patient population extracted for 2009 to 2018 and 2% in 2019. To match the 2% sample data in 2019 with identical sampling weights, the data between 2010 and 2018 were resampled.

### 2.2. Study design and population

The study population consisted of patients who received a diagnosis of gout (International Classification of Diseases-10 code: M10) as their principal diagnosis between 2010 and 2019. To be included in the analysis, patients were required to have complete data and to have visited a general hospital-tertiary/general hospital/hospital, convalescent hospital, clinic, South Korean medicine hospital, South Korean medicine clinic, or health center/health subcenter/health post/health medical center, regardless of age or sex.

### 2.3. Data analysis

The HIRA-NPS data for patients with gout were classified by age (divided into 7 age groups in 10-year increments from < 20 to ≥ 70 years), sex, payer type (National Health Insurance [NHI], Medicaid, others), type of visits to medical institutions (outpatient, inpatient), and type of medical institutions (general hospital-tertiary/general hospital/hospital, convalescent hospital, clinic, South Korean medicine hospital, South Korean medicine clinic, or health center/health subcenter/health post/health medical center). The percentages in each category were analyzed.

To analyze the cost of care for gout, the number of patients, total costs, and cost per patient were analyzed and converted into US dollars. The annual costs from 2010 to 2019 and the ratio of average annual increase over 10 years were also analyzed. The number of patients per month was analyzed over 10 years, and the data were presented to identify seasonal trends.

Prescribed medications for gout were categorized as NSAIDs, ULTs, gastrointestinal medications, corticosteroids, and other medications. The subcategories of ULTs were analyzed for the 4 most prescribed ULTs: allopurinol, benzbromarone, colchicine, and febuxostat.

Comorbidities were classified as musculoskeletal disorders, respiratory diseases, cardiovascular and metabolic diseases, and other diseases. Musculoskeletal disorders were analyzed in more detail by subdividing the affected area into the trunk, upper extremities, and lower extremities.

All statistical analyses for this study were performed using SAS 9.4 (2002–2012 by SAS Institute Inc., Cary, NC, USA).

### 2.4. Ethics approval

The study protocol was approved by the HIRA Deliberative Committee for public data provision, and the study was conducted in accordance with relevant guidelines and regulations. The study was reviewed and qualified for an exemption by the Institutional Review Board of Jaseng Hospital of Korean Medicine, Seoul, South Korea (JASENG 2023-03-005). Since this study analyzed publicly available data, no consent was obtained from the participants by the authors. All personal information was deidentified prior to public release. The authors had no access to information that could identify individual participants during or after data collection.

## 3. Results

### 3.1. General characteristics of the study population

The HIRA-NPS data included a total of 69,680 patients who visited medical institutions for gout treatment between 2010 and 2019 (Table 1).

**Table 1:** Basic characteristics of patients.

Category		Total (2010–2019)	
		No. of patients	Percentage
Age	<20	371	0.5
	20–29	3633	5.2
	30–39	10,934	15.7
	40–49	15,359	22.0
	50–59	16,978	24.4
	60–69	12,143	17.4
	≤70	10,262	14.7
Sex	Male	63,421	91.0
	Female	6259	9.0
Payer type*	National Health Insurance (NHI)	67,153	96.4
	Medicaid	2401	3.5
	Others	126	0.2

* Others include military and veterans medical institutions.

Most of the patients were above the age of 40 (40–49: 22.0%, 50–59: 24.4%, 60–69: 17.4%, 70–79: 14.7%), and most were men (91.0%). Most patients were covered by the NHI (96.4%), followed by Medicaid at 3.5%.

Of the 301,919 claims in the HIRA-NPS (Table 2), outpatient care accounted for 99.5% of all claims, with only a small percentage of patients receiving inpatient services. General hospital-tertiary/general hospital/hospital accounted for 29.9% of all claims, indicating that patients mainly visited primary healthcare institutions for outpatient care regarding gout management.

**Table 2:** Basic characteristics of medical usage.

Category		Total (2010–2019)	
		No. of patients	Percentage
Type of visit	Outpatient	300,349	99.5
	Inpatient	1570	0.5
Medical institution	General hospital-tertiary/general hospital/hospital	90,299	29.9
	Convalescent hospital	1481	0.5
	Clinic	200,722	66.5
	Korean medicine hospital	325	0.1
	Korean medicine clinic	7430	2.5
	Health center/health subcenter/health post/health medical center	1662	0.6

### 3.2. Frequently prescribed medications

**Figure 1.**
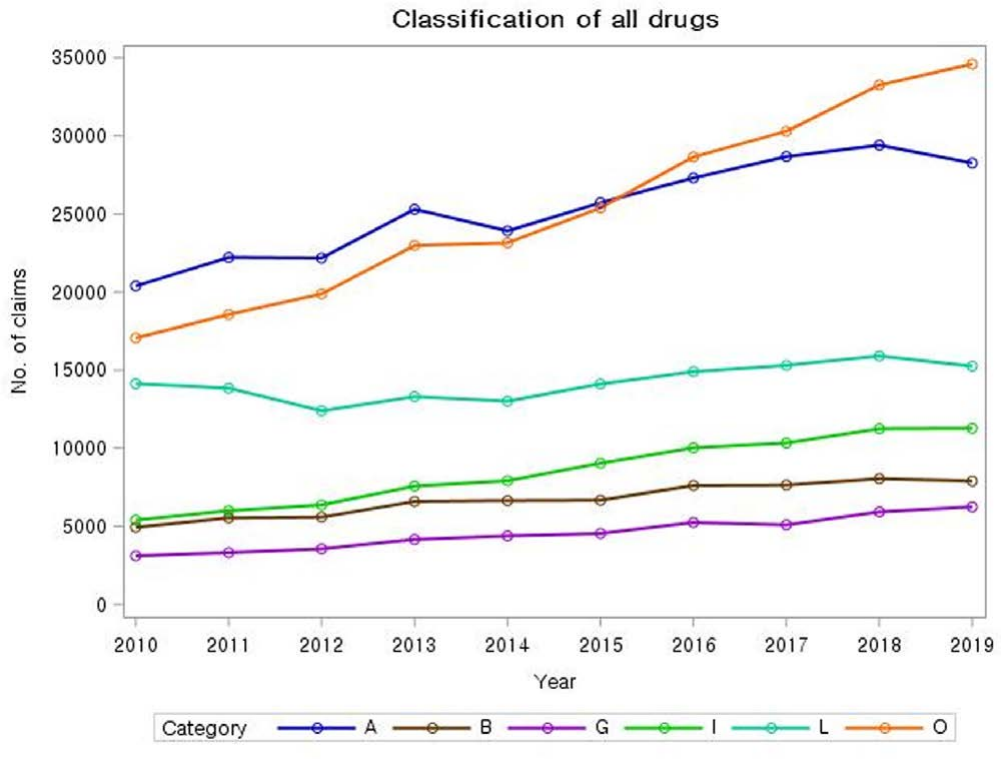
**Classification of all drugs.** Note: (A) NSAIDs, (B) opioid analgesics, (C) antivirals, (D) antiprotozoals/antifungals, (E) antibiotics, (F) antihistamines, (G) cardiovascular medications, (H) diabetes medications, (I) corticosteroids, (J) diagnostic agents, (K) Medications used for surgeries and procedures, (L) gastrointestinal medications, (M) osteoporosis medications, (N) diuretics, (O) urate-lowering treatments (ULTs), and (P) liver disease medications. NSAIDs = nonsteroidal anti-inflammatory drugs.

Figure 1 illustrates the classification of medications and the number of prescriptions for each medication. NSAIDs were prescribed 253,336 times during the entire study period, with a mild increase from 20,386 in 2010 to 28,254 in 2019. ULTs were prescribed 253,802 times during the entire study period, with the largest increase from 17,055 cases in 2010 to 34,594 cases in 2019. Since 2016, the number of ULT prescriptions has exceeded the number of NSAID prescriptions, making ULTs the most used medication for patients with gout. Gastrointestinal medications were prescribed for 142,131 cases, corticosteroids for 85,109 cases, opioid analgesics for 67,100 cases, cardiovascular medications for 45,476 cases, and antihistamines for 5574 cases.

**Figure 2.**
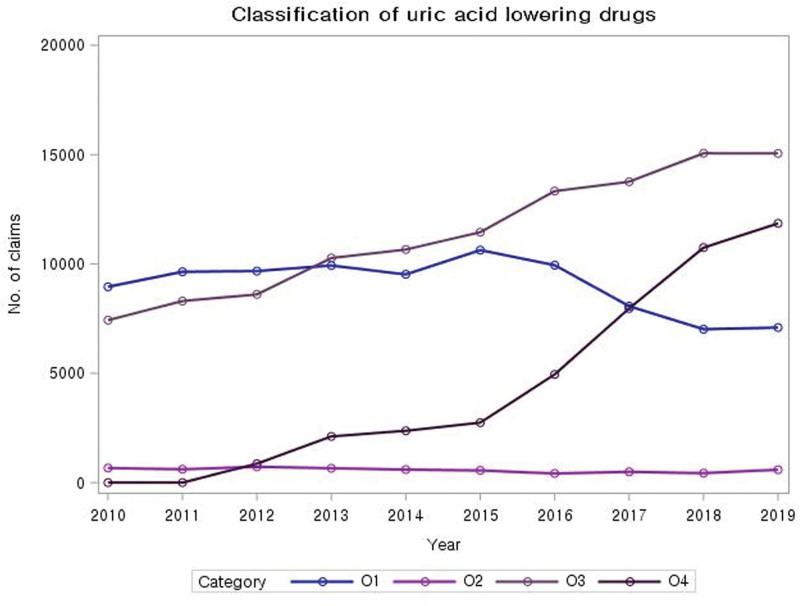
**Classification of uric acid lowering drugs.** Note: O1: allopurinol, O2: benzbromarone, O3: colchicine, O4: febuxostat.

Allopurinol, benzbromarone, colchicine, and febuxostat were the most frequently used ULTs (Fig. 2). Colchicine was the most prescribed ULT, accounting for 113,944 cases. Allopurinol was the most prescribed medicine until 2012; however, since 2013, the number of colchicine prescriptions has surpassed that of allopurinol prescriptions. Febuxostat was first administered in 868 cases in 2012; its use demonstrated a progressive increase, with 10,748 cases prescribed in 2018. Consequently, febuxostat became the second most prescribed ULT, surpassing allopurinol. Benzbromarone was the least commonly prescribed medication among the 4 ULTs, with only 5749 prescriptions between 2010 and 2019.

### 3.3. Cost

Table 3 displays the trends in the number of patients, total costs, and cost per patient from 2010 to 2019. The incidence of gout grew from 4700 in 2010 to 9454 in 2019, representing an average yearly growth rate of 8.07%. Total costs increased from $437,525 in 2010 to $1020,725 in 2019, with an average annual growth rate of 9.87%. This percentage increase is greater than that in the number of patients. The cost per patient increased from $93 in 2010 to $120 in 2014 before falling to $108 in 2019. The average annual growth rate for the cost per patient over the decade (2010–2019) was 1.66%.

**Table 3:** Cost structure.

Category	Yr										
	2010	2011	2012	2013	2014	2015	2016	2017	2018	2019	CAGR
No. of patients	4700	5286	5541	6297	6448	7036	7735	8187	8996	9454	8.07%
Total costs ($)	437,525	558,494	575,064	697,360	772,919	775,810	855,785	902,659	1020,885	1020,759	9.87%
Cost per patient ($)	93	106	104	111	120	110	111	110	113	108	1.66%

### 3.4. Seasonality

**Figure 3.**
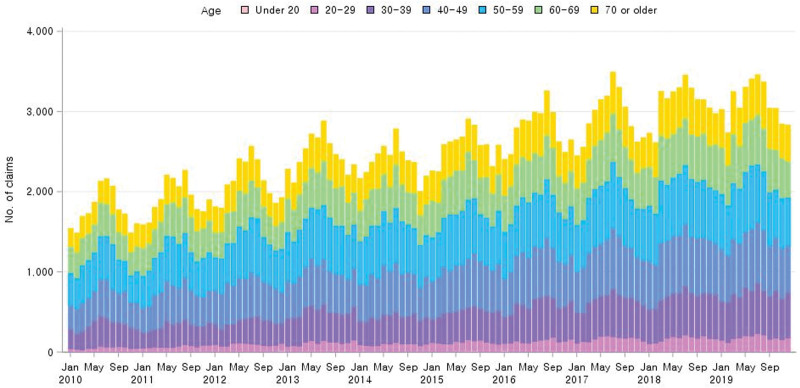
**Number of monthly claims with a main diagnosis of gout between 2010 and 2019.** To examine the seasonal trend, the number of claims with a diagnosis of gout was analyzed by mo for the 10 yr from 2010 to 2019.

To investigate the possible correlation between gout incidence and seasonal change, we visualized the trend in the number of claims from 2010 to 2019. While the number of claims demonstrated a trend of yearly increase, the number of gout claims was highest in May to August, after which the number of claims decreased and was the lowest in November to February (Fig. 3). When analyzing the increasing trend of gout claims by age group, no one age group exhibited a significant increase in claims; rather, an increase was observed across all age groups.

### 3.5. Comorbidity

**Figure 4.**
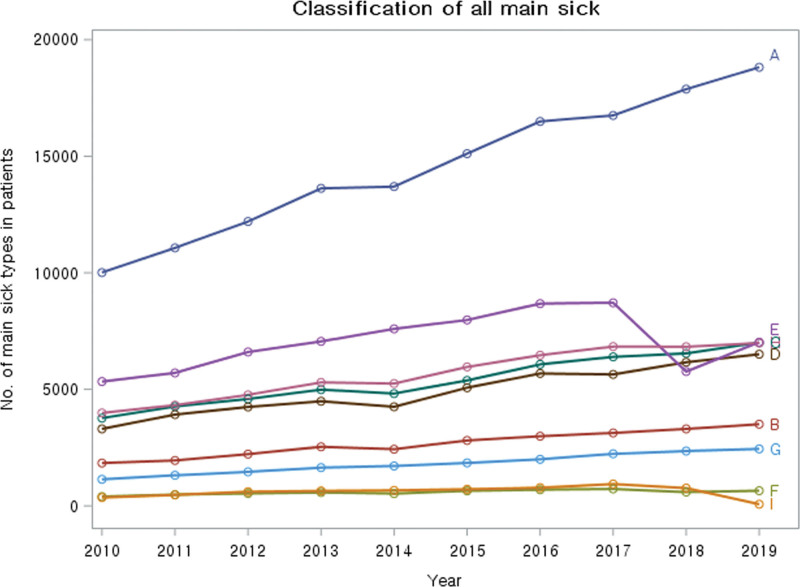
**Classification of all main sick.** Note: (A) musculoskeletal, (B) immune system, (C) cardiovascular system and metabolism, (D) gastrointestinal system, (E) respiratory system, (F) liver, pancreas, and gallbladder, (G) kidney and urinary system, (H) Ophthalmology, otolaryngology, and dermatology, (I) neuropsychiatry.

The distribution of the comorbidities of patients who visited medical institutions with gout was examined (Fig. 4). The most common comorbidity was musculoskeletal disorders, with 145,654 cases during 2010 to 2019; this condition steadily increased from 10,010 cases in 2010 to 18,820 cases in 2019. Respiratory diseases were the second most common comorbidity, with 70,444 cases, followed by cardiovascular and metabolic diseases, with 53,816 cases.

**Figure 5.**
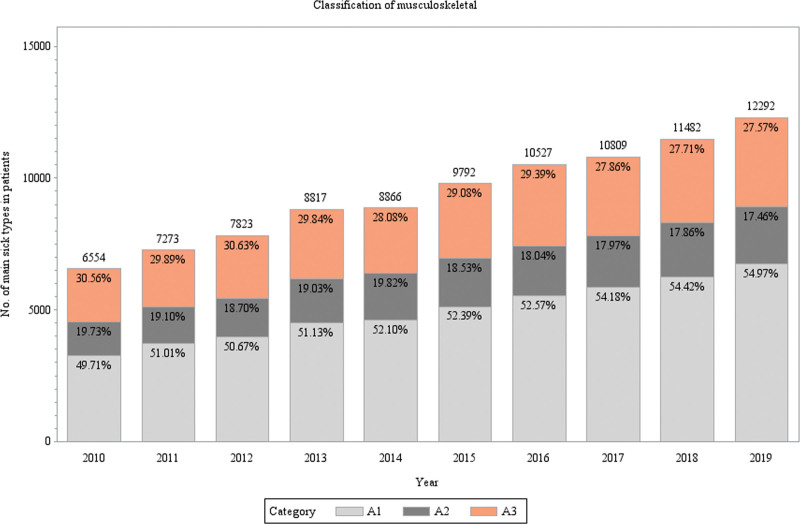
**Classification of musculoskeletal disorders (A1: trunk, A2: upper extremities, A3: lower extremities).** A more detailed analysis of musculoskeletal disorders was performed by classifying the affected area into trunk, upper extremities, and lower extremities.

Figure 5 illustrates the comorbid musculoskeletal illnesses categorized by the affected area. The prevalence of musculoskeletal disorders of the trunk grew from 49.71% in 2010 to 54.99% in 2019 during the study period. The proportion of lower extremities affected by musculoskeletal diseases fell from 30.56% in 2010 to 27.57% in 2019, whereas the proportion of upper extremities affected declined from 19.73% in 2010 to 17.48% in 2019.

## 4. Discussion

Most patients sought outpatient care for gout management, and most were covered by the NHI, indicating the importance of primary healthcare institutions in managing this condition. NSAIDs and ULT were the most prescribed medications, with a significant increase in the use of colchicine and febuxostat among ULTs. The high prevalence of musculoskeletal problems as comorbidities suggests the importance of addressing these conditions in conjunction with gout treatment. Seasonal trends demonstrated an increase in gout claims from June to August, possibly owing to lifestyle factors such as increased alcohol consumption and dietary habits during the summer months. The total cost of treating gout increased over the 10 years, with a higher rate of increase than that in the number of patients; however, the cost per patient did not increase significantly. Notably, gout is more prevalent among men because of several factors, including hormonal differences and lifestyle factors such as increased alcohol consumption and meat intake. As for the higher prevalence of gout among middle-aged and older adults, it is likely because of an increase in the risk factors associated with aging, such as decreased kidney function and changes in connective tissue. Therefore, addressing these risk factors in addition to gout treatment can be beneficial for overall health outcomes.^[10]^

The most frequently prescribed medications for gout patients were NSAIDs, accounting for 28.9% of prescriptions; ULTs, accounting for 29%; gastrointestinal medications, accounting for 16.2%; and corticosteroids, accounting for 9.7%. Although gastrointestinal medications are not directly linked to gout, they have often been prescribed as concomitant medications to prevent gastrointestinal disturbances that may be caused by other medications. Interestingly, since 2016, ULT prescriptions have outnumbered those of NSAIDs, which could reflect the current trend of treating gout as a chronic disorder requiring long-term management rather than merely focusing on treating acute gout attacks.^[11]^

While perceptions on ULTs have been mixed due to its effectiveness and risks for adverse effects, recent studies^[12]^ pointed out that low-dose ULTs are just as effective as high-dose, and advised its use for consistent gout flares which do not respond to NSAIDs and corticosteroids. Correspondingly, recent clinical practice guidelines recommend low-dose colchicine as one of the first-line treatments for acute gout attacks. Additionally, it is recommended that ULTs be initiated early in the course of the disease for every patient diagnosed with gout,^[13,14]^ allowing for an effective management of gout with less risk of side effects.

This study observed a significant change in the trend of prescribing ULTs. Allopurinol was the most prescribed medication in 2010, but its use gradually decreased over the years. By contrast, prescriptions of colchicine and febuxostat increased and outnumbered allopurinol by 2019. Colchicine was the most frequently prescribed ULT, accounting for 44.9% of all ULTs prescribed during the study period, whereas febuxostat was first prescribed in 2012, and its prescription rate demonstrated a sharp increase. As a first-generation ULT, allopurinol has been associated with a risk of side effects, such as renal impairment, especially when used in high doses.^[15]^ This has resulted in a gradual decrease in the number of prescriptions. Colchicine is known to be primarily effective in stabilizing acute flares, making it the most frequently prescribed ULT. Febuxostat has demonstrated greater effectiveness with fewer side effects than allopurinol in many large-scale randomized controlled trials, which could explain the rapid increase in its use for gout management.^[16]^ The trend in ULT prescriptions in South Korea, as observed in this study, is consistent with the consensus among clinicians that ULTs should be used cautiously.^[17]^

A previous study reported increased direct and indirect gout-related disease burdens in Europe.^[18]^ In the present study, the direct costs associated with gout treatment in South Korea exhibited an average yearly increase of 9.87% from 2010 to 2019, indicating that the social burden of gout in South Korea is also rising in terms of total costs. However, the results demonstrated that the rate of change in cost burden per patient did not significantly increase, with an average annual increase rate of 1.66%. This can be attributed to the increase in the number of patients receiving long-term management, as gout is now considered a chronic condition.

Moreover, based on national sample data, this study revealed a seasonal trend of high gout incidence in summer. Although previous studies have reported mixed results regarding the seasonal trend in gout prevalence, some suggest that gout attacks are more common during the summer months rather than the winter months.^[19–21]^ Interestingly, a previous study examined Google Trends and reported that the keyword “gout” is the most searched term during summer in South Korea.^[22]^ Additionally, a previous multicenter study conducted in South Korea reported similar findings, where the incidence of gout was the highest in the summer months (June, July, and August), and ULTs were most prescribed in autumn. However, this study had some limitations, including the small sample size and potential selection bias.^[23]^ Notably, various factors known to affect gout, such as age, sex, purine intake, medication use, and comorbidities, may also be influenced by seasonal changes. Therefore, the relationship between gout and seasonal variations may be multifactorial and requires further research.

The most common comorbidities observed among gout patients were musculoskeletal, respiratory, and cardiovascular and metabolic diseases. The continuously increasing trends in cardiovascular and metabolic diseases, gastrointestinal diseases, and ophthalmological, otolaryngological, and dermatological diseases indicate the healthcare utilization patterns of patients over the age of 40 with chronic diseases. When musculoskeletal diagnoses were categorized by affected body regions, the trunk was the most affected area, with chronic lower back pain being a common example. Although gout attacks typically cause pain in the toes, musculoskeletal disorders in the upper and lower extremities demonstrated a gradual decline over the study period. While there are no specific guidelines considering musculoskeletal comorbidity of gout patients, future strategies may require such perspectives to address patients’ chronic symptoms.

The current study implicates the importance of the role of primary healthcare institutions and caregivers and their use of medications for the management of gout and its comorbidity. Recent clinical guidelines^[12,14,24,25]^ strongly recommend low-dose colchicine, NSAIDs, or glucocorticoids as first-line therapy for gout flares, and ULT after 2 to 4 weeks after a gout flare has settled. Updates from clinical guidelines can be beneficial especially to primary caregivers who are often involved with long-term management of gout patients. Furthermore, recent studies indicate the importance of keeping in mind the safety of certain gout medications on risks of comorbidity, cardiovascular safety^[26]^ and with aging.^[27]^

The present study has several limitations. First, as the HIRA-NPS database only includes items covered by NHI, medical service utilization in actual clinical settings may have been underestimated. Second, as the analysis focused on the total number of prescriptions, it was difficult to determine trends in combined therapy versus monotherapy prescriptions. In fact, according to clinical practice guidelines on the treatment of gout,^[14,28]^ prescriptions of combined therapies account for a considerably higher proportion compared with monotherapy prescriptions, which was not considered in this study. Additionally, this study was limited by its cross-sectional design, allowing only 1 year of follow-up for each patient. Although the number of claims for comorbidities was analyzed, detailed and accurate trends in comorbidity could not be fully elucidated owing to long-term follow-up constraints.

Despite some limitations, such as a cross-sectional approach and lack of long-term follow-up, this study makes a significant contribution to the understanding of gout management and treatment in South Korea. Further research is necessary to explore the multifactorial relationship between gout and seasonal changes and improve the accuracy of comorbidity trends.

## 5. Conclusion

The present study sheds light on the healthcare utilization and management of gout in South Korea, utilizing comprehensive NPS data spanning a decade backed by claims data provided by the HIRA. By analyzing patient characteristics, medication trends, comorbidities, costs, and seasonality, this study provides valuable evidence for healthcare policymakers to improve gout management and treatment. In particular, as the patient sample was based on the entire South Korean population, this study results may serve as a reference for clinicians in choosing appropriate medications and management strategies for gout patients. Moreover, the finding of a seasonal trend in the incidence of gout in summer could aid in the development of national health policies and budget planning for the disease. Further research is required to address the seasonality of gout patients and related management strategies.

## Author contribution

**Conceptualization:** Do-Hyun Kang, Ye-Seul Lee.

**Data curation:** Ye-Seul Lee.

**Formal analysis:** Ye-Seul Lee.

**Supervision:** In-Hyuk Ha, Ho Seub Song.

**Writing – original draft:** Do-Hyun Kang, Ye-Seul Lee.

**Writing – review & editing:** Yoon Jae Lee, In-Hyuk Ha.
